# Effect of home-based transcranial direct current stimulation (tDCS) on cognitive functioning in bipolar depression

**DOI:** 10.21203/rs.3.rs-5396838/v1

**Published:** 2024-12-10

**Authors:** Hakimeh Rezaei, Rachel D. Woodham, Ali-Reza Ghazi-Noori, Philipp Ritter, Elvira Bramon, Michael Bauer, Allan H. Young, Cynthia H.Y. Fu

**Affiliations:** TU Dresden; University of East London; University of East London; TU Dresden; University College London; TU Dresden; King’s College London; University of East London

**Keywords:** Transcranial direct current stimulation, bipolar depression, cognitive impairment, home-based treatment, Neuropsychological Tests

## Abstract

Bipolar depression is commonly accompanied by cognitive impairments. Transcranial direct current stimulation (tDCS) is emerging as a novel non-invasive treatment for bipolar depression. Given the portability and safety of tDCS, we developed a home-based protocol with real-time supervision. Our aim was to assess the cognitive effects of tDCS in bipolar disorder. 44 participants (31 women, mean age 47.27 ± 12.89 years) experiencing a moderate to severe depressive episode received 21 sessions of home-based tDCS over 6 weeks. The stimulation protocol involved 2 mA in a bilateral frontal montage (F3 anode, F4 cathode) for 30 minutes per session. The cognitive assessments were conducted at baseline and after the course of treatment. Rey Auditory Verbal Learning Test (RAVLT) was used to assess verbal learning and memory and the Symbol Digit Modalities Test (SDMT) to assess psychomotor processing speed and visuospatial attention. 93.18% (n=41) completed RAVLT and 59.09% of participants (n=26) completed SDMT. A significant improvement was observed in the RAVLT short-term verbal memory score post-treatment (p = 0.002). Improvements were observed in RAVLT verbal learning, RAVLT post-interference recall, and SDMT, which were not statistically significant. In summary, cognitive performance showed an improvement in bipolar depression following treatment with home-based tDCS, suggesting it can be an effective intervention for cognitive deficits in bipolar depression and positively impact cognitive function.

## 1. Introduction

Cognitive impairments are a significant feature in bipolar disorder. Impairments are observed in all states of bipolar disorder, including the euthymic phase (Minichino et al., 2015), affecting social and occupational functioning (Baune and Malhi 2015). Among these, deficits in verbal learning and memory, psychomotor speed, executive function, and sustained attention are the most commonly observed (Keramatian et al. 2021). Bipolar depression is usually more common and longer lasting than the manic episode (Belmaker and Bersudsky 2004). Studies indicate that depressive symptoms have a greater impact on functional impairment than hypo(manic) symptoms in individuals with bipolar disorder (Rosa et al. 2010).

Various treatment approaches have been explored to improve cognitive performance in individuals with bipolar disorder. Pharmacological interventions, such as lithium and **antipsychotics** like **quetiapine** and **risperidone**, show mixed results. Lithium, while initially associated with cognitive slowing effects in attention and memory, may have fewer long-term negative effects and in some cases, may help preserve cognitive function over time. Antipsychotics like quetiapine can negatively impact psychomotor speed, attention, and working memory. **Valproate** similarly shows mixed effects on **memory** and **executive function**. Lamotrigine, however, improves verbal fluency and memory (Sanches et al. 2015). Pharmacological agents, such as lurasidone, an antipsychotic, and **erythropoietin**, a hormone with neuroprotective effects, have demonstrated some cognitive benefits, particularly in improving working and visual memory, although the long-term effects of erythropoietin are less clear (Tamura et al., 2021). Mifepristone, a glucocorticoid receptor antagonist, has shown positive effects on spatial working memory and verbal fluency (Young et al. 2004). Non-pharmacological interventions, such as cognitive remediation therapy (CRT) and non-invasive brain stimulation techniques have shown potential in enhancing attention, memory, and executive function, but further research is needed to confirm their effectiveness (Solé et al. 2017).

Transcranial direct current stimulation (tDCS) is a novel non-invasive technique used to modulate brain activity by delivering a weak current (1–2 mA) to the scalp via two electrodes (anode and cathode) (Nitsche et al. 2008). Meta-analyses of randomized sham-controlled trials have shown that tDCS is effective in achieving response and remission in major and bipolar depression compared to sham treatments (Mutz et al., 2018). Research findings on the cognitive effects of tDCS in individuals with bipolar disorder have been mixed and remain relatively limited. Improvement has been reported in verbal learning and memory (Minichino et al., 2015; Bersani et al., 2017; McClintock et al., 2020) as well as selective attention and information processing speed (McClintock et al. 2020) following tDCS sessions. However, a study observed no improvement in working memory and sustained attention following tDCS treatment compared to a sham condition (Martin et al., 2015).

Until recently, tDCS trials had administrated the treatment in a clinic or research centre, which requires frequent visits to a clinical setting, creating barriers to access. Due to its portability and safety, tDCS can be administered at home. We have developed a home-based tDCS treatment protocol that is self-administered by participants with real-time remote supervision by researchers using video conference, targeting the key impairments associated with bipolar depression. Our randomised controlled trial of home-based tDCS in unipolar major depressive episode has shown significant clinical improvements, safety, and high acceptability (Woodham et al. 2024). We anticipated improvement in cognitive functioning in bipolar depression.

The validity and acceptability of web-based neuropsychological testing is increasingly supported by recent research. Such tests have proven effective across various populations, including healthy older adults (Cyr et al., 2021), clinical groups, such as individuals with Parkinson’s disease (Binoy et al. 2023), bipolar disorder (Miskowiak et al. 2021), and multiple sclerosis (Eilam-Stock et al. 2021). They often produce results that positively correlate with those obtained from traditional pen-and-paper tests, making them an increasingly popular choice in both research and clinical settings (Gualtieri and Johnson 2006).

To date, there is no existing research on the impact of home-based tDCS treatment with remote supervision on cognitive functioning in individuals with bipolar depression. In the current study, verbal learning and memory were assessed using the Rey Auditory Verbal Learning Test (RAVLT) (Rey 1964), which evaluates how well an individual can learn, retain, and recall verbal information across multiple trials and time delays. Psychomotor processing speed and visuospatial attention were assessed using the Symbol Digit Modalities Test (SDMT) (Smith 1991). We aimed to investigate change in performance over time and to compare cognitive functioning to a control group of participants.

## 2. Materials and methods

### 2.1. Study design and tDCS protocol

Participants were recruited via online advertisements, secondary care, and GP referrals. After study details were explained and any questions were answered, informed written consent was obtained electronically. Ethical approval was granted by the London Fulham Research Ethics Committee.

The study was an open-label, single-arm acceptability and feasibility trial of home-based tDCS for bipolar depression (ClinicalTrials.gov: NCT05436613 registered on 23 June 2022 https://www.clinicaltrials.gov/study/NCT05436613). The protocol involved 30 minutes of active tDCS with a bifrontal montage: anode at left DLPFC (F3) and cathode at right DLPFC (F4), delivered 5 times a week for 3 weeks, then 2 times a week for another 3 weeks, totaling 21 sessions. A minimum of 15 sessions was required. The stimulation was set at 2 mA for 30 minutes with a with a gradual ramp up over 120 seconds at the start and ramp down over 15 seconds at the end of each session. The Flow Neuroscience© tDCS device was used with real-time remote supervision from research team member via Microsoft Teams.

Healthy controls were recruited through online ads or local outreach and completed baseline study activities and did not have tDCS stimulation.

### 2.2. Inclusion and exclusion criteria

Inclusion criteria for participants with bipolar depression were adults aged 18 or older with a Diagnostic Statistic Manual of Mental Disorders, Fifth Edition (DSM-5) (American Psychiatric Association 2013) diagnosis, confirmed by the Mini-International Neuropsychiatric Interview (MINI; Version 7.0.2) (Sheehan et al. 1998), experiencing a current depressive episode with a Montgomery-Åsberg Depression Rating Scale (MADRS) score of 18 or higher (Montgomery and Åsberg 1979). Participants had to be on a stable dose of mood stabilizers or medication-free for at least two weeks.

Exclusion criteria included concurrent psychiatric disorders, significant suicide risk, a Young Mania Rating Scale (YMRS) score greater than 8 (Young et al. 1978), scalp or skin conditions, metallic implants, epilepsy or seizures with loss of consciousness, neurological disorders, or migraines.

Healthy controls had to be at least 18 years old and were excluded if they had a personal or family history of psychiatric disorders, obsessive-compulsive disorder, significant suicide risk, or a YMRS score greater than 8.

### 2.3. Neuropsychological assessments

Two neuropsychological assessments were conducted at baseline and after the 6-week treatment period, with the order and versions counterbalanced. IQ was measured using the Ammons Quick Test(Ammons and Ammons, 1962).

The RAVLT was used to assess verbal learning and memory. The test involved two lists (A and B) of 15 words each. List A was read aloud at one-second intervals, and participants were instructed to immediately recall as many words as possible. This procedure was repeated for five consecutive trials. After the fifth trial, List B, an interference list, was presented in the same manner. Following List B, participants were asked to recall as many words as they could from List A (Trial 6). We calculated the following RAVLT scores: total learning (sum scores of trials 1 to 5), which assessed the short-term verbal memory; learning over trials (subtracting five times the score of Trial 1 from the sum of the scores of trials 1 to 5), measuring of verbal learning; and short-term percentage retention (total of Trial 6 divided by Trial 5, expressed as a percentage), assessing post-interference recall.

The SDMT was used to evaluate information processing speed, psychomotor speed, and visuospatial attention. Participants were mailed the two paper versions of the test and were provided with clear instructions during the study visit video conference. In this test, participants encountered a grid displaying nine symbols paired with the numbers one to nine which were displayed below the symbols. In a larger grid on the same side of paper, symbols were shown but without corresponding numbers in the spaces below. The task was to write the missing numbers in the empty spaces using the reference grid. Participants were asked to record the missing values in the spaces provided without skipping any symbols. The primary goal was to complete as many correct matches as possible within 90 seconds. The results were recorded by capturing screenshots directly after the task was completed.

### 2.4. Statistical analysis

Comparison of demographic variables between the treatment group and the control group consisted of independent sample t-tests for continuous variables (age, years of education and IQ), and chi-square test for categorical variable (gender).

Repeated measures ANOVAs were conducted to evaluate neuropsychological scores for bipolar depression participants, with RAVLT and SDMT scores as the dependent variables and assessment time-point as the within-subjects factor, with two levels including baseline and end of treatment period. To assess the differences in neuropsychological features between bipolar depression and control participants, independent samples t-tests were conducted. Statistical analyses were conducted using IBM SPSS for Mac version 29.0. All analyses were two tailed and a significance value of p = 0.05 was set.

## 3. Result

### 3.1. Participants

The demographic and clinical features of the bipolar depression and healthy control groups are presented in Table 1. The bipolar depression group had significantly higher MADRS scores at baseline compared to healthy control participants (p > 0.001). There were no other significant differences in demographic features between the groups.

A total of 44 bipolar depression participants (31 women) were enrolled, with a mean age of 47.2 ± 12.94 years and a baseline mean MADRS score of 24.6 ± 2.64. 93.2% of participants (n=41) completed the full 6-week course of treatment.

All 41 participants completed the RAVLT at week 6, with a mean age of 47.93 ± 13.15 and a mean MADRS score of 24.95 ± 3.11. 59.09%. 26 participants completed the written version of the SDMT at baseline and at week 6, with a mean age of 51.42 ± 10.38 and a mean MADRS of 25.08 ± 3.68.

The control group consisted of 28 healthy adults (17 women) with a mean age of 44.68 ± 14.45 years and a mean MADRS score of 0.75 ± 1.07.

### 3.2. Neuropsychological assessments

**Figure 1:**
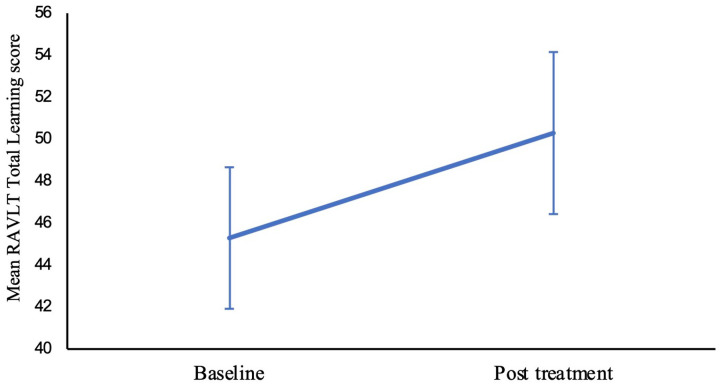
Changes in mean RAVLT total learning score from baseline to post-treatment

Neuropsychological assessment results for bipolar depression participants are presented in Table 2. At week 6, the mean RAVLT total learning score for bipolar depression participants improved significantly from 45.29 ± 11.00 at baseline to 50.29 ± 12.62 at week 6 (F _(1, 40)_ = 10.672, p = .002) (Figure 1). The mean RAVLT learning over trial score increased from 16.63 ± 6.31 at baseline to 17.73 ± 8.54 at week 6 (F _(1, 40)_ = 0.475, p = 0.495), and short-term percentage retention scores rose from 83.16 ± 21.65 to 84.45 ± 16.17 (F (_1, 40_) = 0.108, p = 0.744), however, these improvements were not statistically significant. There was a trend towards a significant difference in SDMT scores from baseline (mean = 44.35 ± 19.94) to week 6 (mean = 46.88 ± 11.01) (F (_1, 25_) = 3.28, p = .082).

In the healthy control group, mean RAVLT total learning score was 51.50 ± 10.8, mean learning over trial score was 16.32 ± 8.77, mean short-term percentage retention score was 90.81 ± 11.01, and mean SDMT score was 46.88 ± 11.01. Baseline scores were lower in the bipolar depression group relative to healthy controls, however, the difference was not statistically significant (Table 3).

## 4. Discussion

Cognitive impairment in bipolar disorder affects daily functioning and quality of life, especially during depressive episodes. This study explored the impact of home-based tDCS with real-time remote supervision on cognitive functioning in BD. Home-based tDCS was well tolerated, with mild, transient side effects (tingling, redness, itching) and no serious adverse events or mood switching (Ghazi-Noori et al., 2024).

Outcomes indicate that repeated sessions of home-based tDCS can improve cognitive performance in bipolar depression. The significant improvement in RAVLT total learning scores after the course of treatment indicates that the individuals’ short-term memory of verbal information has greatly improved. Additionally, improvements were also noted in RAVLT measures of verbal learning and post-interference recall, as well as psychomotor processing speed and visuospatial attention in SDMT tasks. Although the observed cognitive improvements were slight, they still suggest a positive trend that may be clinically relevant. Contrary to our expectation, we did not observe a significantly poorer performance for bipolar depression group at baseline compared to the healthy control group.

The current findings align with studies showing that tDCS can improve verbal learning and memory, selective visual attention, auditory attention, and information processing speed and executive functioning in individuals with bipolar depression (McClintock et al. 2020), as well as in euthymic bipolar patients (Minichino et al., 2015; Bersani et al., 2017; McClintock et al., 2020). Improvements in **SDMT** performance have been observed in studies of both **unipolar** (Fregni et al. 2006) and **bipolar depression** (McClintock et al. 2020). However, some studies reported no significant **improvements** in RAVLT and SDMT after tDCS treatment compared to sham conditions in patients with **major depressive disorder** (Martin et al. 2018) and **bipolar depression**, despite initial SDMT improvements observed only in the latter (Loo et al. 2012). Similarly, Woodham et al. (2024; 2022), under a similar home-based protocol with real-time remote supervision, found no changes in **RAVLT** and **SDMT** scores among individuals with **MDD**. These results suggest that while tDCS may enhance cognitive function, its effectiveness in improving cognitive deficits in bipolar disorder warrants further investigation.

When compared to other treatments, tDCS has demonstrated more consistent cognitive improvements (Solé et al. 2017). **Pharmacological treatments** such as **lithium** and **antipsychotics** (e.g., **quetiapine**) often show **mixed results**, with cognitive impairments commonly reported, particularly in **verbal memory** and **psychomotor speed** (Sanches et al., 2015). Tamura et al. (2021) evaluated the effects of **erythropoietin**, a neuroprotective hormone, in bipolar depression, finding improvements in **working and verbal memory** as measured by RAVLT. However, they also reported that these cognitive benefits diminished over time.

A limitation of this study is the absence of a sham treatment arm, as all participants received active tDCS in an open-label design. Additionally, the small sample sizes for RAVLT and SDMT might have been underpowered and limit the generalization of the findings. Another limitation is the **lack of long-term assessment** to determine whether the cognitive improvements observed following **repeated tDCS sessions** are sustained over time. To minimize potential delays and distractions associated with audio-based testing, we opted for the written version of the SDMT.

## 5. Conclusion

A 6-week course of home-based tDCS can enhance verbal learning and memory performance in patients with bipolar depression. Psychomotor processing speed and visuospatial attention also improved however, this change was not significant. These findings underscore the need for further investigation in studies with larger sample sizes and the use of randomized controlled trials. While tDCS shows promise as a non-invasive intervention for cognitive enhancement in BD, additional research is required to determine its long-term effects.

## Figures and Tables

**Table 1.** Demographic and clinical data at baseline

	BD group	BD RAVLT completers	BD SDMT completers	HC group
**Total number (Female)**	44 (31)	41 (29)	26 (26)	28 (17)
**Mean Age (years)**	47.27 ± 12.9	47.93 ± 13.15	51.42 ± 10.38	44.68 ± 14.45
**Age range (years)**	24–76	24–76	30–76	21–72
**Years of education**	16.30 ± 2.46	16.37 ± 2.66	16.27 ± 292	16.89 ± 2.11
**IQ**	100.66 ± 9.3	101.29 ± 9.31	102.77 ± 9.38	103.39 ± 8.77
**MADRS**	24.59 ± 2.64	24.95 ± 3.11	25.08 ± 3.68	0.75 ± 1.07
**Duration of illness (years)**	18.98 ± 12.47	19.02 ± 11.33	21.85 ± 11.4	
**Duration current depressive episode (weeks)**	49.55 ± 100.4	50.68 ± 103.8	63.62 ± 126.9	
**Previous number of episodes**	18.16 ± 16.13	21.39 ± 24.13	24 ± 28.1	

Mean values are presented with ± standard deviation. BD, bipolar depression; HC, healthy control; RAVLT, Rey Auditory Verbal Learning Test; SDMT, Symbol Digit Modalities Test; MADRS, Montgomery-Åsberg Depression Rating Scale.

**Table 2.** Neuropsychological test scores for bipolar depression participants at baseline and after the course of treatment

	Baseline	Post-treatment	F-value	P-value
**RAVLT_TL**	45.29 ± 11.00	50.29 ± 12.62	10.672	0.002
**RAVLT_LOT**	16.63 ± 6.31	17.73 ± 8.54	0.475	0.495
**RAVLT_STPR**	83.16 ± 21.65	84.45 ± 16.17	0.108	0.744
**SDMT**	44.35 ± 19.94	46.88 ± 11.01	3.281	0.082

Mean values are presented with ±standard deviation. BD, bipolar depression; RAVLT, Rey Auditory Verbal Learning Test; RAVLT_TL, total learning; RAVLT_LOT, learning over trials; RAVLT_STPR, short-term percentage retention; SDMT, Symbol Digit Modalities Test. RAVLT (n=41), SDMT (n=26).

**Table 3.** Neuropsychological test scores for bipolar depression participants and healthy control participants at baseline

	BD	HC	F-value	P-value
**RAVLT_TL**	45.29 ± 11.00	51.50 ± 10.80	0.074	0.024
**RAVLT_LOT**	16.63 ± 6.31	16.32 ± 8.77	0.935	0.864
**RAVLT_STPR**	83.16 ± 21.65	90.81 ± 11	5.050	0.090
**SDMT**	44.35 ± 19.94	47.15 ± 8.49	0.305	0.302

Mean values are presented with ±standard deviation. BD, bipolar depression; HC, healthy control; RAVLT, Rey Auditory Verbal Learning Test; RAVLT_TL, total learning; RAVLT_LOT, learning over trials; RAVLT_STPR, short-term percentage retention; SDMT, Symbol Digit Modalities Test. RAVLT (n=41), SDMT (n=26), HC (n=28).

## Data Availability

The anonymised datasets used and/or analysed during the current study are available from the corresponding author on reasonable request.

## Abbreviations

*BD*: Bipolar Depression
CRT: Cognitive Remediation Therapy
*DSM-5*: Diagnostic Statistic Manual of Mental Disorders, Fifth Edition
DLPFC: Dorsolateral Prefrontal Cortex
HC: Healthy Control
mA: milliAmpere
*MADRS*: Montgomery-Åsberg Depression Rating Scale
MDD: Major Depressive Disorder
*MINI*: Mini-International Neuropsychiatric Interview
RAVLT: Rey Auditory Verbal Learning Test
RAVLT_TL: Rey Auditory Verbal Learning Test-Total Learning
RAVLT_LOT: Rey Auditory Verbal Learning Test-Learning Over Trial
RAVLT_STPR: Rey Auditory Verbal Learning Test-Short-Term Percentage Retention
rTMS: repetitive Transcranial Magnetic Stimulation
SDMT: Symbol Digit Modalities Test
*tDCS*: transcranial direct current stimulation
